# Design and rationale of a randomized–controlled trial of home-delivered meals for the management of symptomatic ascites: the SALTYFOOD trial

**DOI:** 10.1093/gastro/goz005

**Published:** 2019-03-11

**Authors:** Elliot B Tapper, Jad Baki, Scott Hummel, Anna Lok

**Affiliations:** 1 Division of Gastroenterology and Hepatology, Department of Internal Medicine, University of Michigan, Ann Arbor, Michigan, USA; 2 University of Michigan, Ann Arbor, Michigan, USA; 3 Division of Cardiology, Department of Internal Medicine University of Michigan, Ann Arbor, Michigan, USA

**Keywords:** Readmissions, malnutrition, cirrhosis, nutrition, sodium

## Abstract

**Background:**

When patients with cirrhosis develop ascites, it is associated with sharply increased mortality and healthcare utilization with decreased quality of life. Dietary salt restriction is first-line therapy for ascites but it is limited by poor adherence.

**Methods:**

We will recruit 40 patients with cirrhosis and ascites who have received a recent paracentesis or hospitalization for a 1:1 randomized trial of standard care (education on salt restriction) versus home-delivered meals. Our primary outcome is the number of paracenteses needed over 12 weeks. Secondary outcomes include hospital-bed days, health-related quality of life (HRQOL, Ascites Symptom Inventory-7 and Visual Analogue Scale) and performance on batteries of physical function including hand grip (kg) and walk speed (m/s). All subjects follow up through a series of calls where any paracenteses, hospital readmissions, weight changes and diuretic dosage changes are recorded. In a final Week 12 visit, knowledge of dietary sodium intake, quality of life and frailty are reassessed, and satisfaction with the meal-delivery program is evaluated. Paired comparison testing will be conducted between the two arms.

**Discussion:**

A nutritionally standardized meal-delivery program for patients with cirrhosis and ascites post discharge has a variety of potential patient-based benefits, including the effective management of ascites, reduction of healthcare utilization and improvement of HRQOL. We have three core hypotheses. First, patients will report interest in and satisfaction with a home-delivered meals program. Second, subjects on a salt-restricted (2 g sodium) meal-delivery program will have fewer therapeutic paracenteses and all-cause readmissions than subjects receiving standard of care. Third, subjects on a salt-restricted (2 g sodium) meal-delivery program will report increased HRQOL compared to subjects receiving standard of care.

## Background

The prevalence of liver disease and cirrhosis in the USA has almost doubled in the last decade, resulting in a substantial rise in associated morbidity and mortality [1, 2]. When a person with cirrhosis develops a clinical decompensation, the median survival drops precipitously from 10–20 years for persons with compensated cirrhosis to less than 2 years [2]. Ascites, occurring in as many as 50% of patients with cirrhosis, is associated with life-threatening infections, renal dysfunction, malnutrition, diminished health-related quality of life (HRQOL) [3] and frequent hospitalizations. Efforts to reduce patients’ ascites burden have cascading positive effects on well-being, freedom from morbidity and healthcare costs. However, the effective management of ascites can be very challenging.

High salt consumption leads to increased water retention and quicker build-up of ascites. Therefore, first-line therapy for ascites is dietary salt restriction (2,000 mg/d [88 mmol/d]) [4]. Diuretic therapy is a useful adjunct for the management of ascites but it is also associated with significant side effects (e.g. renal injury, hyper/hypokalemia, hyponatremia). Efforts to educate patients (and caregivers) on the health benefits of salt restriction, the current standard of care, are important components of high-quality care for patients with cirrhosis, but have an unclear impact [5]. Although it is effective, adherence to salt restriction is very poor, which may have contributed to the negative results of some randomized, albeit dated, trials of salt restriction [6, 7].

Patients with cirrhosis pose unique challenges to trials of salt restriction. First, the intended effects of diuretics can interfere with social and daily function, jeopardizing adherence. Second, many patients with cirrhosis are socially isolated, often lacking the adequate social support necessary to encourage adherence to an extended salt-restricted diet protocol.

One solution to the problem of poor salt-restriction adherence is to have patients consume nutritionally standardized home-delivered meals. Home-delivered meals have been linked to reductions in expected readmissions and the incidence of falls for nutritionally at-risk persons [8]. In a recent randomized–controlled trial (GOURMET-HF), patients with acutely decompensated congestive heart failure were randomized to receive home-delivered low-salt meals or usual care [8]. Diet adherence was average in that trial, with subjects reporting that about 75% of meals consumed were the study-approved salt-restricted meals. Subjects on the meal-delivery arm of that trial had, on average, fewer hospitalizations, as well as improved symptoms [9].

Beyond controlling salt intake, home-delivered meals could be broadly effective for multiple reasons in patients with cirrhosis. First, many patients with cirrhosis are malnourished owing in part to food insecurity or a lack of education on optimal diet. Ascites worsens malnutrition, causing anorexia and exacerbating catabolic processes. Second, many patients with ascites have comorbid hepatic encephalopathy, which worsens in the context of malnutrition and improves with adequate calorie/protein intake [10]. Malnutrition and hepatic encephalopathy lead to sarcopenia and cognitive dysfunction, which can manifest as physical frailty [11]. Frailty, in turn, is associated with increased mortality [11]. Third, ascites has significant adverse effects on quality of life (QOL) independent of other indices of disease severity [3, 12]. For these reasons, we hypothesized that both ascites control and improved nutrition may be key to a host of important patient-centered outcomes.

## Aims and hypotheses

***Aim 1***: To determine the feasibility of a larger, multicenter meal-delivery program for patients with cirrhosis and ascites.

***Hypothesis 1***: Patients will report interest in and satisfaction with a home-delivered meals program.

***Aim 2***: To determine the effect size of a salt-restricted (2 g sodium) meal-delivery program with respect to reducing the need for therapeutic paracenteses and all-cause readmissions for patients with cirrhosis and ascites compared to subjects receiving the standard of care.

***Hypothesis 2***: Subjects on a salt-restricted (2 g sodium) meal-delivery program will have fewer therapeutic paracenteses and all-cause readmissions than subjects receiving standard of care.

***Aim 3***: To determine the effect size of a salt-restricted (2 g sodium) meal-delivery program in improving HRQOL for patients with cirrhosis and ascites.

***Hypothesis 3***: Subjects on a salt-restricted (2 g sodium) meal-delivery program will report increased HRQOL compared to subjects receiving standard of care.

## Methods

### General design

This is a randomized–controlled pilot trial to determine the efficacy and feasibility of a pre-prepared, home-delivered, salt-restricted (1,500 mg/d), protein-rich (>80 g/d) meal-delivery program in reducing the need for hospital readmission and therapeutic paracentesis and increasing QOL in patients with cirrhosis and ascites. Subjects are randomized to receive this intervention or a standard-of-care low-salt education pamphlet. Outcomes are detailed in **Table 1**. This trial is registered at ClinicalTrials.gov (NCT03493204). All subjects consented to participate and this study was approved by the University of Michigan Health System IRB (HUM00141457).

**Table 1.** Study outcomes and corresponding data-collection methods

Outcome	Data-collection method
**Primary endpoint**	**Primary outcome data collection**
Therapeutic paracenteses over 12 weeks	Self-reported during follow-up and chart review
**Secondary endpoints**	**Secondary outcome data collection**
Hospital-bed days in 12 weeks	Self-reported during follow-up and chart review
Changes in diuretic dosages	Self-reported during follow-up and chart review
Changes in ascites-related QOL	ASI-7 during baseline and Week 12 visit
Changes in QOL	VAS during baseline and Week 12 visit
Changes in frailty	Hand grip and walk speed during baseline and Week 12 visit

QOL, quality of life; ASI-7, Ascites Symptom Inventory-7; VAS, Visual Analogue Scale.

### Intervention

Subjects in the standard-of-care arm only receive a standardized educational pamphlet with information on how to follow a lower-sodium (2,000 mg/d sodium) diet. Those randomized to the meal-delivery arm receive this same pamphlet and are also placed on a salt-restricted diet (nutritional targets of 1500–2,000 mg/d sodium, >80 g/d protein and 1,500–2,000 kCal/d) for 28 days. First, this diet includes 84 home-delivered meals prepared by PurFoods, LLC (Ankeny, IA) in consultation with dietitians. Each meal (average of 553 kilocalories and 18 g protein) comes from a salt-restricted menu that meets the aforementioned nutritional targets. Subjects eat three meals a day for 28 days. Second, subjects are given a 1-month supply (30 servings) of ProCel Whey Protein Powder from which they will consume one serving (15 g protein, 110 calories, 105 mg sodium per serving) each night. Third, subjects are asked to purchase milk (any brand of their choosing) and consume three servings daily (6–8 g protein, 100 calories and 100 mg sodium per serving).

### Study procedures

#### Baseline examination

All demographics, socioeconomic details, comorbidities, alcohol use, activities of daily living, fall history and measures of disease severity are recorded (Child Class, MELD). QOL is assessed by the Visual Analogue Scale of Quality of Life (VAS) and the Ascites Symptom Inventory-7 (ASI-7) scales. The VAS is a scale from 0 (indicating the worst health imaginable) to 100 (indicating the best health imaginable). The ASI-7 is a validated scale for ascites-specific HRQOL [12]. Frailty is assessed in two ways: hand grip (kg) and 10-m walk speed (m/s). The extent of the subject’s current knowledge of dietary sodium intake is evaluated using a questionnaire.

#### Interventions

Subjects are randomized using sealed envelopes to evenly (1:1) distribute subjects between two arms (a meal-delivery arm and a standard-of-care arm). All patients receive phone calls at Weeks 1, 2, 4 and 8 to record any hospitalizations, paracenteses, changes to diuretic dosages and current weight.

#### Final Week 12 visit

The sodium knowledge questionnaire is reassessed, as are measures of frailty (hand grip and walk speed) and QOL (ASI-7, VAS).

## Discussion

 The personal and economic burden of ascites complicating cirrhosis is severe. Further, given that the clinical treatment of symptomatic ascites is risky—therapeutic paracentesis (with costs and risks) or hospitalization (profoundly costly and risky)—the benefits of an intervention to reduce the risk of symptomatic ascites may justify an upfront investment.

Readmissions involving patients with cirrhosis are common (more than one in every four discharges) [13], morbid and costly. Symptomatic ascites is a major reason for readmission [14]. Interventions proven to prevent hospitalization for symptomatic ascites are lacking. Further, no prior intervention has sought to improve ascites through home-delivered meals. The trade-offs of our proposed intervention are favorable. Two people can be fed through our intervention for 28 days for the price of 1 hospital-bed day. Further, hospitalization can be dangerous by exposing vulnerable patients to the risk of complications such as infections, falls and by confining them to a bed, worsening their already compromised physical and nutritional status.

## Home-delivered meals may have global benefits

**Figure 1.**
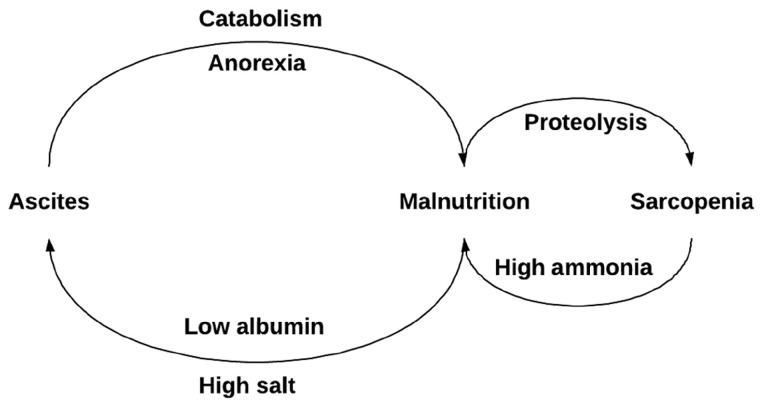
The benefits of optimized ascites through nutrition: a conceptual model. Poor nutrition is central to the pathophysiology of cirrhotic complications. Poor nutritional intake, characterized by inadequate protein and high salt, drives ascites. Ascites worsens nutritional status by causing anorexia and increased resting energy expenditure (catabolism). Malnutrition, in turn, leads to proteolysis and sarcopenia. Given that muscle is central to ammonia metabolism, lower skeletal muscle mass increases ammonia levels. Hyperammonemia contributes to hepatic encephalopathy, which may disrupt dietary habits.

Malnutrition is a central process in the pathophysiology of cirrhosis complications, as shown in **Figure 1**. Protein-energy malnutrition is associated with reduced survival in patients with cirrhosis. Ascites can become infected—an event that is often fatal in the short term and can also cause renal failure. Ascites causes anorexia and is a catabolic process that results in progressive sarcopenia. Sarcopenia, in turn, is associated with frailty and hepatic encephalopathy, both of which are factors that sharply increase the mortality of cirrhosis. Control of ascites may forestall the development of malnutrition. In this trial, we attempt to reverse malnutrition by decreasing abdominal distension and supplement protein. Further, we are encouraging night-time snacking with a high-protein supplement to address impaired hepatic gluconeogenesis in patients with cirrhosis. A randomized trial has shown that a high-protein night-time snack prevents catabolism during overnight ‘fast’ and improves total body protein [15]. For these reasons, salt restriction and protein supplementation may have more general benefits than ascites control.

## Limitations and mitigation of anticipated issues

First, as with any study examining long-term effects of salt-restricted diet on patient outcomes, one inherent limitation is imperfect diet adherence by subjects in the meal-delivery arm. While the European Association for the Study of the Liver recommends 4.6–6.9 g/d sodium in patients with ascites [16], we recognize that our salt-restricted diet may be less palatable. However, we will track satisfaction prospectively, adjusting the foods delivered to suit the tastes of the patient. For outcomes to change with home-delivered meals, adherence to the provided meals and protein supplementation must be high and ingestion of high-salt food outside the protocol must be low. Second, given the clinical tenuousness of decompensated cirrhosis, participation may be unexpectedly put on hold or terminated. This is a key focus for determination of this feasibility trial.

## Conclusions and future directions

The goal of the SALTYFOOD trial is to provide proof-of-principle evidence to support the design of a larger, multicenter trial in a larger cohort. Our goal is to determine the efficacy and long-term impact not just on ascites control, but also on QOL, admissions and survival. Given the upfront investment needed, estimates of cost-effectiveness will be important to justify widespread adoption.

## Authors’ contributions

E.B.T. conceived of and designed the project. E.B.T. and J.B. collected the data. E.B.T., J.B., S.H. and A.L. analysed and interpreted the data. E.B.T. and J.B. drafted the manuscript. S.H. and A.L. made critical revisions. All authors read and approved the final manuscript.

## Funding

E.B.T. receives funding from the National Institutes of Health through the Michigan Institute for Clinical and Health Research (KL2TR002241). The content is solely the responsibility of the authors and does not necessarily represent the official views of the National Institutes of Health. The funder played no role in the design or execution of the study.
