# Design and rationale of a randomized–controlled trial of home-delivered meals for the management of symptomatic ascites: the SALTYFOOD trial

**DOI:** 10.1093/gastro/goz017

**Published:** 2019-04-13

**Authors:** Elliot B Tapper, Jad Baki, Scott Hummel, Anna Lok

**Affiliations:** 1 Division of Gastroenterology and Hepatology, Department of Internal Medicine, University of Michigan, Ann Arbor, Michigan, USA; 2 University of Michigan, Ann Arbor, Michigan, USA; 3 Division of Cardiology, Department of Internal Medicine University of Michigan, Ann Arbor, Michigan, USA

*Gastroenterology Report*, 2019;7:146–9. https://doi.org/10.1093/gastro/goz005

The original version of this article contained a mistake in the authors' affiliations; this has now been corrected. The publisher would like to apologize for this mistake.

